# Reduced expression of chemerin is associated with poor clinical outcome in acute myeloid leukemia

**DOI:** 10.18632/oncotarget.21440

**Published:** 2017-09-30

**Authors:** Jing Zhang, Jiao Zhou, Xi Tang, Ling-Yu Zhou, Ling-Ling Zhai, Minse Evola-Deniz Vanessa, Jing Yi, Yun-Yun Yi, Jiang Lin, Jun Qian, Zhao-Qun Deng

**Affiliations:** ^1^ Department of Laboratory Center, The Affiliated People's Hospital of Jiangsu University, Zhenjiang, 212002, Jiangsu, People's Republic of China; ^2^ Department of Hematology, The Affiliated People's Hospital of Jiangsu University, Zhenjiang, 212002, Jiangsu, People's Republic of China

**Keywords:** chemerin, diagnosis, prognosis, acute myeloid leukemia, biomarker

## Abstract

Chemerin is dysregulation in numerous solid cancers. However, only little is known about the role of chemerin in acute myeloid leukemia (AML). In this study, we aimed to investigate the expression and clinical significance of recently described chemerin in acute myeloid leukemia (AML). The expression of chemerin in 149 patients with de novo AML and 35 normal controls was quantified by Real-time quantitative PCR (RQ-PCR). Chemerin was down-expressed in AML compared with controls (P=0.042). A receiver operating characteristic (ROC) curve revealed that chemerin expression could differentiate patients with AML from control subjects (AUC=0.611, 95% CI: 0.490-0.732; P=0.042) respectively. The cohort of AML patients was divided into two groups according to the cut-off value of 0.0826 (79% sensitivity and 54% specificity, respectively). In addition, the AML patients with low chemerin expression had significantly shorter overall survival (OS) than those with high chemerin expression (P=0.049). Moreover, multivariate survival analysis confirmed that chemerin was an independent prognostic factor for AML patients. In conclusion, downregulation of chemerin might be a useful diagnostic and prognostic factor for AML patients.

## INTRODUCTION

Acute myeloid leukemia (AML), the most common type of adult leukemia, is characterized by the accumulation of cloning and differentiation arrest in the bone marrow and blood. It is easy to cause fatal infection, bleeding, or organ infiltration [1]. Acute myeloid leukemia with myelodysplasia-related changes is identified by brief morphologic, cytogenetic and clinical features, patients with this disease still have significant heterogeneity in clinical behavior and response to treatment [2–5].

Chemerin (RARRES2 [retinoic acid receptor responder 2] and TIG2 [tazarotene induced gene 2]) [6], is purified from the ascetic fluids of ovarian cancer patients and has been shown to be a natural ligand for G protein-coupled receptor-1 (GPR-1) and chemokine C-C motif receptor-like-2 (CCRL-2) [7, 8]. Certainly, the role of chemerin as a chemoattractant is to promote the recruitment of these cells into sites of tissue injury and lymphoid organs [9, 10]. Chemerin is found to be highly expressed in a variety of tissues, such as adipose tissue, liver, pancreas, skin, etc, which regulates the function of innate immune cells [11, 12]. Growing evidence suggests that chemerin might also have a role in cancer development. Some studies have revealed that the expression of chemerin is different in some types of cancers. Chemerin expression was significantly decreased in hepatocellular carcinoma (HCC) [13], skin squamous cell carcinoma [14], melanoma [15] compared with normal and/or benign tumors in each organ. Conversely, another study showed that the expression of chemerin was overexpressed in colorectal cancer [16], squamous cell carcinoma of the oral tongue [17], gastric cancer [18] correlated with tumor angiogenesis and poor clinical outcomes of patients and upregulatedin grade III/IV glioma tissues compared with grade II ones or brain samples from patients with epilepsy [19]. Nevertheless, these surveys demonstrate that the dysregulation of chemerin may have an important impact on tumorigenesis and progression, but the expression and roles of chemerin in AML remain unclear.

In this study, we aimed to investigate chemerin expression levels and its predictive role in de novo AML patients, and explored its relationship with clinical parameters. It could provide clinical diagnostic and prognostic biomarker for AML.

## RESULTS

### Chemerin expression in normal controls and AML patients

**Figure 1:**
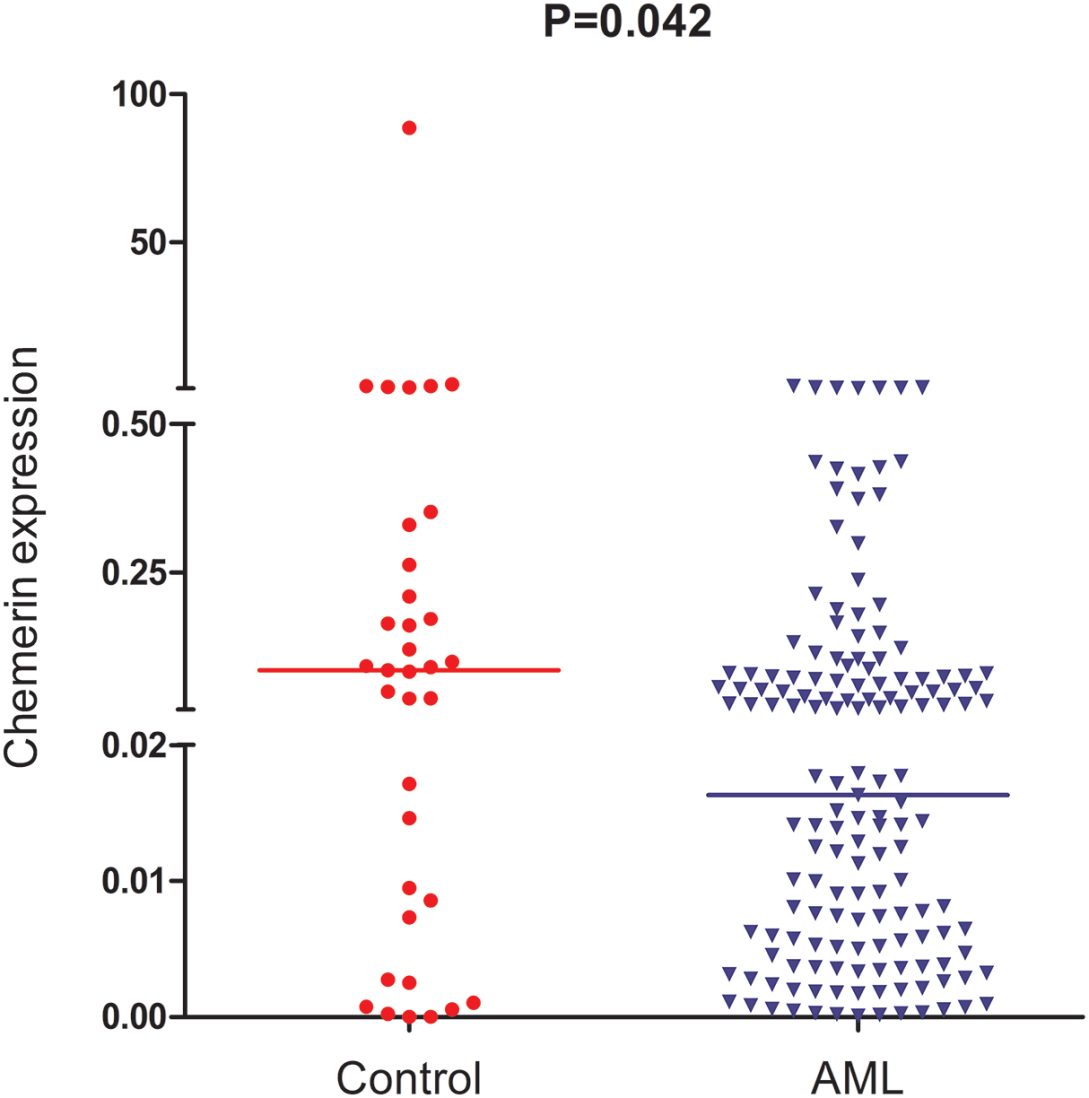
Relative expression levels of chemerin in AML patients and controls

We detected the level of chemerin expression in AML and normol controls. As shown in Figure 1, the levels of chemerin were significantly decreased in AML patients (0.0000-1.3786, median 0.0151) compared with healthy controls (0.0000-88.5733, median 0.0855, P=0.042).

### Differentiating capacity of chemerin expression

**Figure 2:**
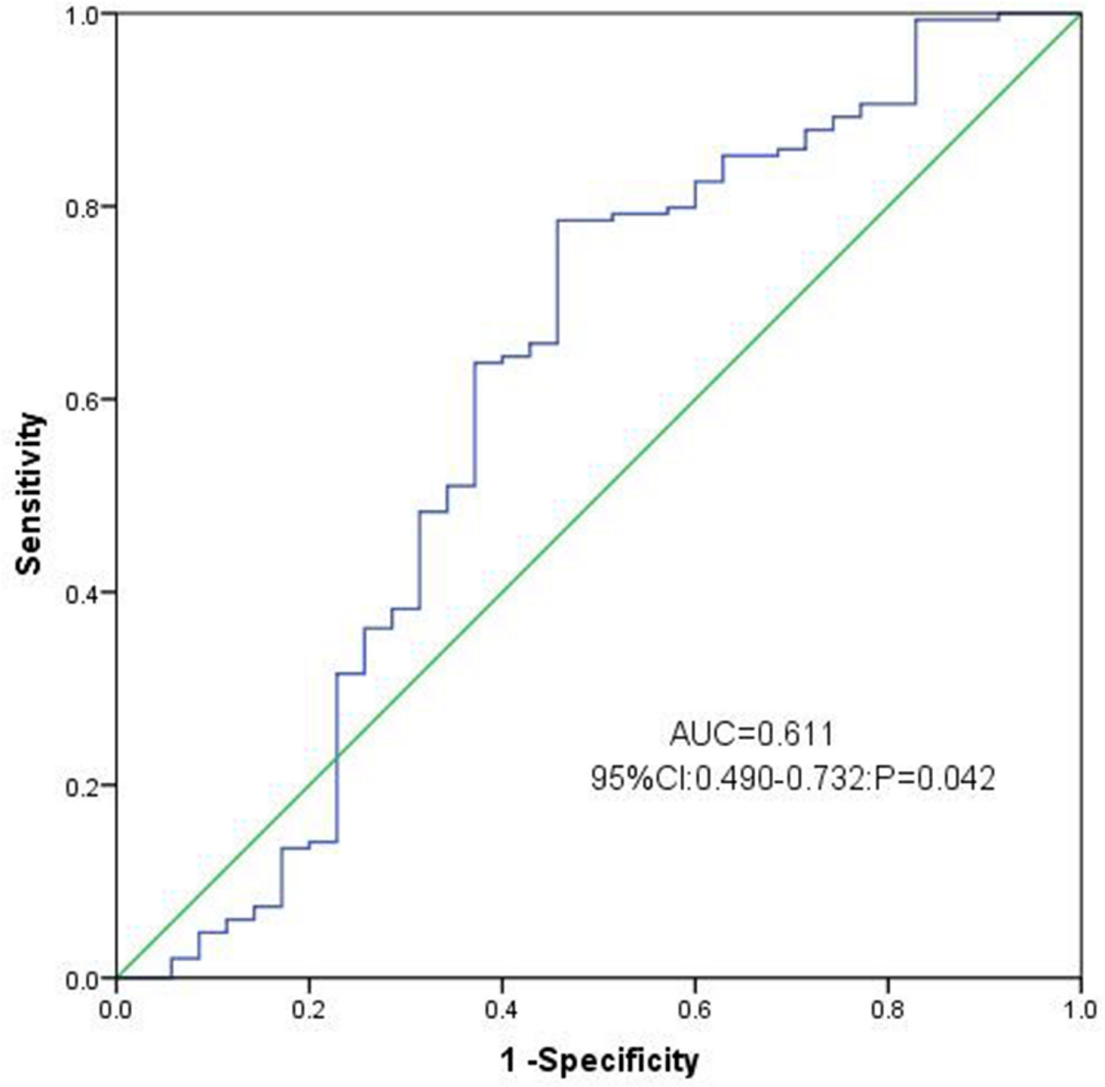
ROC curve analysis using Chemerin for discriminating AML patients from controls

To assess the performance of chemerin expression as a marker, ROC curves were constructed to analyze the sensitivity of this marker in distinguishing AML patients from healthy controls. (AUC=0.611, 95% CI: 0.490-0.732, P=0.042, Figure 2). With a cut-off value of 0.0826, the sensitivity and the specificity were 79% and 54%, respectively. These results demonstrated that chemerin expression might serve as a valuable biomarker for AML diagnosis.

### Clinical and laboratory characteristics of AML patients

To assess the prognostic significance of chemerin expression levels, we used chemerin expression cut-off value 0.0826 as a threshold for dividing 149 AML patients into two groups, high chemerin expression group (≥ 0.0826) and low chemerin expression group (<0.0826). There were no significant differences in age, white blood cells (WBC), hemoglobin (HB), platelets (PLT), and ten gene mutations (DNMT3A, U2AF1, IDH1/2, N/K-RAS, C/EBPA, NPM1 and c-KIT) between chemerin low-expressed group and high-expressed group (P>0.05, Table 1). Moreover, we did not observe significant differences in BM blasts, FAB classifications, and karyotypes (Table 1).

**Table 1:** Comparison of clinical manifestations and laboratory features between AML patients with low and high expression

Patient's parameters	High (n=32)	Low (n=117)	*P* value
Sex, male/female	20/12	68/49	0.690
Median age, years (range)	56.5 (21-80)	56 (10-93)	0.633
Median WBC, ×10^9^/L (range)	9.3 (0.3-154.0)	15.4 (4.0-528.0)	0.424
Median hemoglobin, g/L (range)	75.0 (32-120)	78.0 (41-138)	0.118
Median platelets, ×10^9^/L (range)	33.0 (4-415)	40.0 (3-447)	0.536
BM blasts, % (range)	39.0 (1-99)	49.25 (3-97.5)	0.567
CR (-/+)	13/17	69/41	0.063
FAB			0.227
M0	1 (3%)	2 (2%)	
M1	1 (3%)	6 (6%)	
M2	12 (38%)	43 (37%)	
M3	8 (25%)	23 (19%)	
M4	6 (19%)	25 (21%)	
M5	1 (3%)	16 (13%)	
M6	3 (9%)	2 (2%)	
Karyotype classification			0.615
Favorable	11 (34%)	31 (26%)	
Intermediate	18 (57%)	67 (57%)	
Poor	2 (6%)	16 (14%)	
No data	1 (3%)	3 (3%)	
Karyotype			0.924
normal	15 (48%)	48 (41%)	
t (8;21)	3 (9%)	6 (5%)	
t (16;16)	0 (0%)	1 (1%)	
t (15;17)	8 (25%)	22 (18%)	
+8	0 (0%)	5 (4%)	
-5/5q-	0 (0%)	3 (3%)	
-7/7q-	0 (0%)	1 (1%)	
t(9;22)	0 (0%)	1 (1%)	
others	3 (9%)	14 (12%)	
complex	2 (6%)	13 (11%)	
No data	1 (3%)	3 (3%)	
Gene mutation^*^			
* CEBPA* (+/−)	4/23	12/88	0.745
* NPM1* (+/−)	4/23	11/89	0.523
* FLT3*-ITD (+/−)	4/23	12/88	0.745
* c-KIT* (+/−)	0/27	4/96	0.578
* N/K-RAS* (+/−)	0/27	8/92	0.201
* IDH1/2* (+/−)	0/27	2/98	1.000
* DNMT3A* (+/−)	2/25	7/93	1.000
* U2AF1* (+/−)	2/50	3/47	0.287

WBC, white blood cells; FAB, French-American-British classification; AML, acute myeloid leukaemia; CR, complete remission; ^*^, percentage was equal to the number of mutated patients divided by total cases in each group.

### Association between chemerin expression and clinical outcome

**Figure 3:**
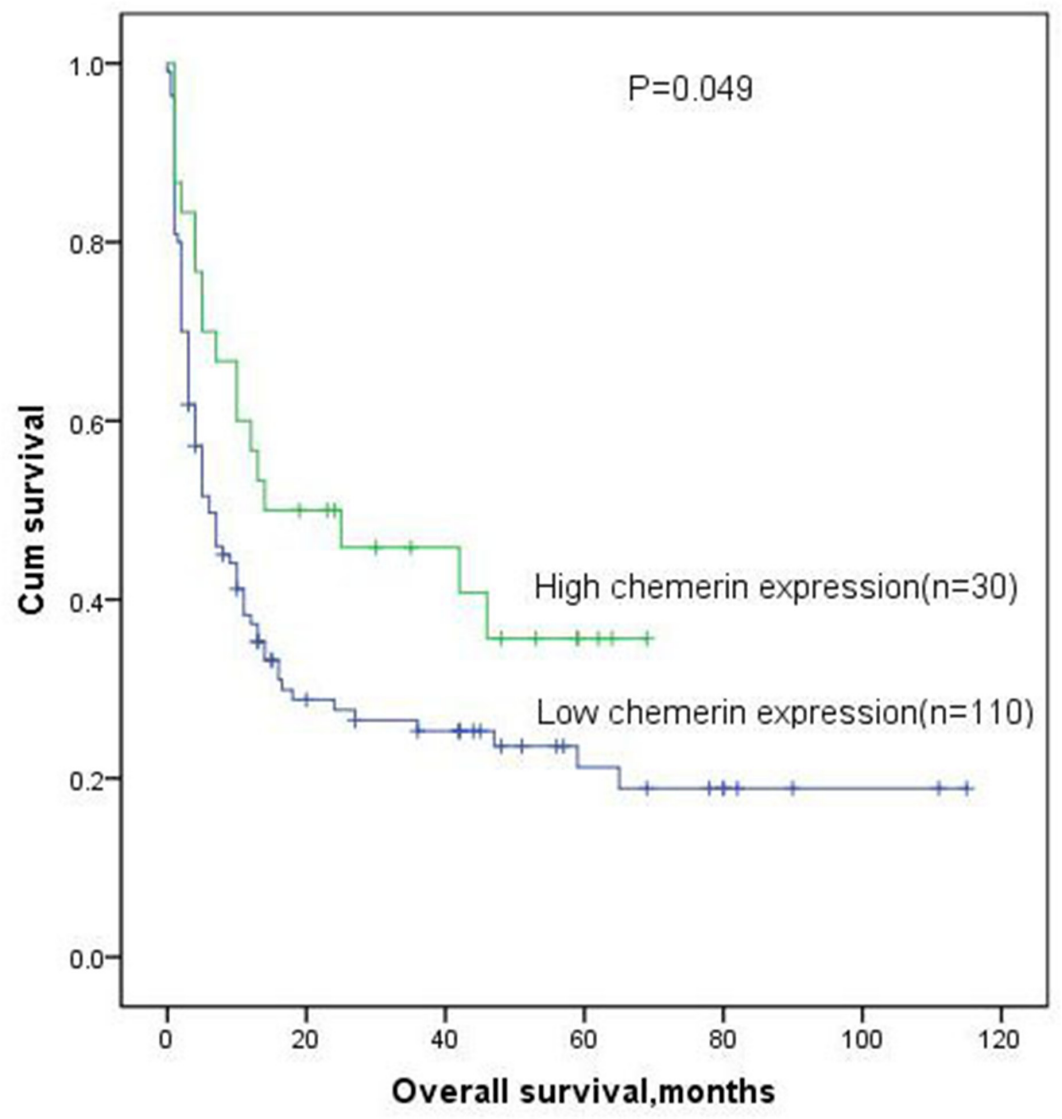
Overall survival analysis of AML patients

Among 149 cases, 140 patients with available follow-up data were eligible for the complete remission (CR) analysis and 9 patients were excluded because of incomplete follow-up. High-expressed patients had no significantly CR rate compared with low-expressed patients in whole AML, however, there was a trend of high chemerin expression toward higher CR after induction therapy (57% vs 41%, P=0.063, Table 1). Kaplan-Meier analysis results showed that patients with low chemerin expression had poorer overall survival (OS) than those with high chemerin expression in whole AML patients (median 5.5 vs 16.5 months, respectively, P=0.049, Figure 3). Multivariate analysis, the parameters associated with age (≥60/<60y), WBC (≥30/<30 ×10^9^/L), karyotype classifications (favorable/intermediate/poor), ten gene mutations (mutant/wild-type), and chemerin expression (high/low) with P<0.20 in univariate analysis, also identified that chemerin over-expression was an independent favorable prognostic factor in AML patients (Table 2).

**Table 2:** Univariate and multivariate analyses of prognostic factors for overall survival in AML patients

	Univariate analysis	*P* value	Multivariate analysis	*P* value
	Hazard ratio (95% CI)		Hazard ratio (95% CI)	
WBC	2.494 (1.809-3.439)	<0.001	1.758 (1.1345-2.726)	0.012
age	3.043 (2.211-4.189)	<0.001	1.842 (1.186-2.860)	0.007
Karyotypic classifications	2.033 (1.685-2.453)	<0.001	1.660 (1.235-2.232)	0.001
*Chemerin* expression(low/high)	1.634 (0.978-2.725)	0.060	0.515 (0.288-0.921)	0.025
*U2AF1* mutation	2.920 (1.348-6.326)	0.007	3.063 (1.173-7.994)	0.022
*FLT3*-ITD mutation	0.875 (0.472-1.621)	0.671	-	-
*NPM1* mutation	1.046 (0.578-1.894)	0.882	-	-
*CEBPA* mutation	1.234 (0.725-2.130)	0.429	-	-
*c-KIT* mutation	0.755 (0.309-1.845)	0.538	-	-
*N/K-RAS* mutation	1.411 (0.690-2.888)	0.346	-	-
*IDH1/2* mutation	0.632 (0.156-2.559)	0.520	-	-
*DNMT3A* mutation	1.341 (0.681-2.640)	0.395	-	-

## DISCUSSION

Chemerin, a novel member of adipokines, is known to be involved in regulating adipogenesis and lipid metabolism, cell proliferation and migration, inflammation and leukocyte trafficking, endothelial angiogenesis and MMP production [7, 20, 21]. Recently, dysregulated expression of chemerin has been observed in numerous solid cancers including hepatocellular carcinoma [13, 22], skin squamous cell carcinoma [14], melanoma [15] and adrenocortical carcinoma [23]. These results suggest that chemerin expression may be tumor-specific in the process of tumorigenesis. However, the expression levels and functions of chemerin in AML have been little known. Our study for the first time reported about chemerin expression and its clinical significance in patients with AML.

In this study, we investigated the expression pattern of chemerin and further analyzed the clinical significance of chemerin expression in de novo AML patients. We provided evidence that chemerin was significantly low-expressed in AML patients compared with the controls. In addition, we investigated by ROC curve analysis, high chemerin expression was a valuable biomarker for discriminating AML from healthy controls. High chemerin expression was observed in 21.5% of AML patients if the cut-off value 0.0826 was used according to ROC curve at the sensitivity of 79% and specificity of 54%. It was shown that chemerin expression may serve as a potential biomarker to distinguish AML patients from normal controls.

Furthermore, our study found that high chemerin expression in whole cohort AML was significantly associated with favorable overall survival. We also demonstrated that the expression of chemerin was an independent prognostic factor for overall survival in de novo AML patients according to multivariate analyses. Interestingly, there was a trend of high chemerin expression toward the higher CR rate, which might be due to the small size of patients with CR in our cohort. These results indicated that the chemerin expression levels was a valuable predictor for the assessment of therapeutic efficacy and status, and might serve as a standard for the therapeutic evaluation in AML.

Chemerin is a chemoattractant for macrophages, NK cells, and dendritic cells that induces cell migration [24, 25]. The chemoattractant effect occurs via the G protein-coupled receptor (GPCR) ChemR23, as well as GPR1, and chemokine (C–C motif) receptor-like 2 (CCRL2) [8, 11]. The chemokine C-C motif receptor-like 2 (CCRL2) expression has been shown on almost all human hematopoietic cells [26]. Previous reports have demonstrated that the chemerin receptor CCRL2 upregulation contributes to glioblastoma cell migration [27], cutaneous squamous cell carcinoma [28], and acute myeloid leukemia [29]. Moreover, to identify the potential roles of Chemerin/CCRL2 axis as a novel therapeutic target and biomarker need further investigations. In gastric carcinomas. Overexpression of chemerin was correlated with advanced clinical stage and enhanced invasiveness of gastric cancer cells. Chemerin was also shown to activate the phosphorylation of p38 and ERK1/2 MAPKs in gastric cancer [18]. Another study showed that Chemerin, depending on the cell type and the receptor expression can activate different subtypes of MAPK (mitogen-activated protein kinase) pathway [28]. Whether chemerin is influence the development of acute myeloid leukemia by activating the MAPK, we need to be further identified. Y Liu-Chittenden et al. indicated that chemerin was low-expressed in adrenocortical carcinoma compared with normal and benign adrenocortical tissues, which was due to epigenetic CpG hypermethylation [23]. It may provide the possibility that CpG hypermethylation is causing chemerin downregulation in AML.

In conclusion, We provide the first expression analysis of chemerin in AML, decreased chemerin expression is negatively correlated with clinical outcome in patients with de novo AML. The chemerin expression was found to be an independent predictive marker in patients with AML. Future experiments will show whether chemerin are potential novel therapeutic targets in AML.

## MATERIALS AND METHODS

### Patients and specimens

A total of 149 patients who underwent primary and curative for de novo AML between 2005 and 2014 at the Affiliated People’ Hospital of Jiangsu University were voted as the study population. Bone marrow samples were collected from 35 healthy donors. The diagnosis and classifications were made according to the French-America-British (FAB) classification and World Health Organization (WHO) criteria [30, 31]. Treatment protocol was described as reported previously [32]. Patients with major clinical parameters, including age, gender, karyotype classification, kayotype, and gene mutation are listed in Table 1.

All patients provided written informed consent. The use of clinical specimens in this study was approved by the Ethic Committee of Affiliated People’ Hospital of Jiangsu University.

### RNA isolation and reverse transcription

The bone marrow mononuclear cells (BMNCs) of AML patients at initial diagnosis and healthy donors were concentrated by Ficoll-Hypaque gradient. Total RNA is extracted through the Trizol reagent (Invitrogen, Carlsbad, CA, USA) according to the manufacturer's instructions. 200 uints of MMLV reverse transcriptase (MBI Fermentas, Hanover, USA) containing 2 μg of total RNA, 10 mM of dNTPs, 10 μM of random hexamer and 80 units of RNAs in were used for reverse transcription on iCycler Thermal Cycler (Eppendorf, Hamburg, Germany) to synthesize cDNA. The system of reverse transcription was incubated for 10 minutes at 25°C, for 60 minutes at 42°C, and then stored at -20°C.

### Real-time quantitative PCR

Real-time quantitative PCR (RQ-PCR) analysis was carried out on a 7500 Thermocycler (Applied Biosystems, CA, USA). RQ-PCR for the final reaction volume of 20μL for each sample consisted of 20ng cDNA, 0.8μM primer, 10μM AceQ qPCR SYBR Green Master Mix (Vazyme Biotech Co., Piscataway, NJ, USA) and 0.4μM ROX Reference Dye 2 (Invitrogen). The PCR conditions were as following: 95°C for 5 minutes for initial denaturation, followed by 45 cycles at 95°C for 10 seconds for denaturation, 62°C for 30 seconds for annealing, and 72°C for 30 seconds for extension, and 80°C for 32 seconds to collect fluorescence, finally followed by 95°C for 15 s, 60°C for 60 s. Positive and negative controls were included in each assay. Relative to chemerin expression levels were calculated according to the following formula: N_chemerin_ = (E_chemerin_) ^ΔCT chemerin1(control-sample)^ ÷ (E_ABL_) ^ΔCT ABL (control-sample)^ ×1000‰. The parameter efficiency (E) derived from the formula E=10(-1/slope) (the slope referred to CT versus cDNA concentration plot).

### Gene mutation detection

IDH1/IDH2, NPM1 and DNMT3A mutations were detected according to the literatures reported previously [33–36]. Using PCR and high-resolution melting analysis (HRMA) to detect the C-KIT and U2AF1 mutations. All positive samples were confirmed by direct DNA sequencing. C/EBPA and FLT3 internal tandem duplication (ITD) were detected by DNA sequencing.

### Statistical analysis

Statistical analysis was performed using IBM SPSS Statistics (SPSS, Chicago, IL, version 20.0). Comparing the difference of qualitative data between patients groups were analyzed with the Chi-square test and Fisher's exact test. For these comparison analyses between the different study groups, Kruskal-Wallis and Mann-Whitney U test were applied. Receiver operating characteristic (ROC) curve and area under the ROC curve (AUC) were calculated to determine the diagnostic accuracy of chemerin expression in distinguishing AML patients from normal controls. Kaplan-Meier and Multivariate analysis were performed to identify factors associated with chemerin expression on survival respectively. All the analyses, P<0.05 was considered statistically significant.
